# Widening participation – recruitment methods in mental health randomised controlled trials: a qualitative study

**DOI:** 10.1186/s12874-023-02032-1

**Published:** 2023-09-21

**Authors:** Mais Iflaifel, Charlotte L Hall, Heidi R Green, Andrew Willis, Stefan Rennick-Egglestone, Edmund Juszczak, Mark Townsend, Jennifer Martin, Kirsty Sprange

**Affiliations:** 1 Nottingham Clinical Trials Unit, University of Nottingham, Nottingham, UK; 2 NIHR MindTech MedTech Co-operative, Institute of Mental Health, School of Medicine, University of Nottingham, Innovation Park, Triumph Road, Nottingham, UK; 3 NIHR Nottingham Biomedical Research Centre, Institute of Mental Health, University of Nottingham, Innovation Park, Triumph Road, Nottingham, UK; 4 Previously: Health Services Research Unit, University of Aberdeen, Aberdeen, UK; 5 COUCH Health, Manchester, UK; 6 Centre for Ethnic Health Research, Leicester/Diabetes Research Centre, University of Leicester, Leicester, UK; 7 School of Health Sciences, Institute of Mental Health, University of Nottingham, Nottingham, UK; 8 NIHR Evaluation, Trials and Studies Coordinating Centre (NETSCC), Southampton, UK

**Keywords:** Mental health, Recruitment, Randomised controlled trials, Thematic analysis, Stigma, Digital divide, Diversity, Inclusivity

## Abstract

**Background:**

Barriers to mental health research participation are well documented including distrust of services and research; and stigma surrounding mental health. They can contribute to a lack of diversity amongst participants in mental health research, which threatens the generalisability of knowledge. Given the recent widespread use of the internet in medical research, this study aimed to explore the perspectives of key partners on the use of online (e.g. social media) and offline (e.g. in-person) recruitment as an approach to improving diversity in mental health randomised controlled trials (RCTs).

**Methods:**

Face-to-face and online interviews/focus groups with researchers working in mental health and Patient and Public Involvement partners in the United Kingdom. Recordings were transcribed and analysed using a combination of inductive and deductive thematic analysis.

**Results:**

Three focus groups and three interviews were conducted with a total N = 23 participants. Four overarching themes were identified: (1) recruitment reach; (2) Demographic factors that affect selection of recruitment method; (3) safety of technology, and; (4) practical challenges. Five main factors were identified that affect the choice of recruitment method: age, complexity of mental health problem and stigma, cultural and ethnicity differences and digital divide. The use of online methods was considered more accessible to people who may feel stigmatised by their mental health condition and with a benefit of reaching a wider population. However, a common view amongst participants was that online methods require closer data monitoring for quality of responders, are not fully secure and less trustworthy compared to offline methods that enable participants to build relationships with health providers. Funding, staff time and experience, organisational support, and technical issues such as spam or phishing emails were highlighted as practical challenges facing online recruitment. All participants agreed that using a hybrid approach tailored to the population under study is paramount.

**Conclusions:**

This study highlighted the importance of offering a flexible and multifaceted recruitment approach by integrating online with offline methods to support inclusivity and widening participation in mental health research. The findings will be used to develop considerations for researchers designing RCTs to improve recruitment in mental health research.

**Supplementary Information:**

The online version contains supplementary material available at 10.1186/s12874-023-02032-1.

## Background

Lack of diversity in trial samples is a moral, ethical and scientific issue [1, 2]. Homogenous groups can skew findings and impact generalisability to the wider population [3]. Greater inclusivity could result in more robust data to inform decisions in healthcare and treatment innovations, potentially reducing disparity in health outcomes [4]. Health inequalities have come to the forefront during the COVID-19 pandemic, where older adults, people with existing health conditions, and people from minoritised ethnic backgrounds in the UK continue to be disproportionately affected [5].

Despite being the gold standard of research to determine effectiveness/efficacy, randomised controlled trials (RCTs) often struggle with participant recruitment, engagement and retention, particularly for mental health interventions [6]. There are a number of reasons why recruitment to mental health RCTs may be particularly challenging. Clinicians may have concerns about the perceived vulnerability of their patients resulting in issues with decision making because of lack of equipoise or burden of research or concerns regarding consent and the lack of capacity to provide informed consent [7, 8]. Patients may also have concerns about cultural stigma and stigma surrounding mental health [9], or concerns regarding the treatment itself [6]. It is also challenging to recruit a diverse population to mental health RCTs when the socioeconomically disadvantaged population are less likely to attend mental health services in-person [10, 11]. Indeed, mental health service use is proportionately lower for males [12], people from ethnic minority backgrounds [13], older participants or those living in more rural areas [14].

In recognition of the need to reduce disparities in participation in research, the National Institute for Health Research (NIHR) Clinical Research Network commissioned the INCLUDE project to provide a framework for researchers and funders when developing research protocols and includes examples of how to broaden inclusivity [15]. The NIHR also developed their Equality, Diversity and Inclusion (EDI) Strategy 2022–2027 to ensure the implementation of inclusive practice in research, culture and systems [16].

Whilst RCTs of mental health conditions have been traditionally conducted in a clinical face-to-face setting, since the late 1990s, there has been a trend towards online or ‘digital’ RCTs in the field of mental health [17]. Online recruitment strategies, by which we mean any use of Internet technologies to recruit research participants, such as social media, Google search engine advertisements, and other website campaigns [18] offer researchers the opportunity to modify their recruitment materials and strategies based on feedback and engagement with the adverts to allow a targeted strategy to reach specific audiences [19, 20]. In comparison to offline recruitment methods such as in-clinic recruitment, soliciting subjects through mail and telephone using health records and registers, media campaigns, newspaper advertisements, and input during radio and television talks [18], online recruitment may reach communities who are not currently engaged with specialist mental health services. This is likely to be particularly important for conditions where specialist care is only offered at centres typically in large cities [21], or when recruiting ethnic minority communities [22].

Despite these advantages, there is notable concern about the “digital divide”, which in its simplest terms reflects those connected to the internet and those who are not, but more recently is considered to reflect differences in usage and internet skills [23]. For example there is evidence ethnic minorities, compared to white groups, access technology more outside the home and less frequently suggesting digital exclusion through availability [24]. In addition lower socioeconomic status has been associated with greater digital exclusion [25]. It is unclear therefore whether a move to solely online delivery of trial procedures (such as recruitment or the intervention itself) will impact (positively or negatively) on health inequalities.

This study, which is part of a wider project, REcruitment in Mental health trials: broadening the ‘net’, opportunities for INclusivity through online methoDs (RE-MIND) [26], aims to identify and provide considerations for use of online methods in the recruitment of participants into mental health RCTs, with a focus on whether online methods can enhance inclusivity. The aim of this qualitative sub-study was to explore the experiences and perspectives of key partners on the use of online and offline methods to recruit trial participants.

## Methods

### Study design

This sub-study drew on the constructivism paradigm, which emphasises the importance of context in the process of knowledge construction and accumulation [27]. This paradigm shaped our view of what type of knowledge about recruitment methods in RCTs would be of value. A qualitative approach was therefore used to investigate the experiences, opinions and ideas of key partners on the use of online and offline recruitment methods in the UK. Focus groups (FGs) and semi-structured interviews with participants were conducted by the researchers (KS, MI and CLH). FGs were used for several reasons, (1) to maximise time and resources, (2) to identify and clarify views in relation to others who may have a similar lived experience and (3) to support sharing of these ideas and similar or different opinions [28]. As FGs are more difficult to schedule than individual interviews [29], the research team decided to offer interviews as an alternative for practical reasons [30] to capture key partners who found it difficult in terms of availability/preference to attend FGs. FGs and individual interviews have been found to be very similar in their ability to generate unique items [29]. In this study, FGs and interviews drew on a similar technique for collecting data by using open-ended questioning with inductive probing of responses. The triangulation of both methods was used to ensure the completeness and richness of the findings [31] and to have a comprehensive understanding of the accounts of key partners’ knowledge pertaining to recruitment methods. This study followed the reporting guidelines set out in the consolidated criteria for reporting qualitative research (COREQ) [32] (Additional file 1).

### Sample

Typical case sampling [33] of research staff working on mental health research and Patient and Public Involvement (PPI) partners were approached. Selection variables [34] included past/current experience working in mental health research, expertise in designing and conducting mental health RCTs, age, gender and ethnicity. We identified research staff via the NIHR Clinical Research Network (CRN), and the UK Trial Managers Network (UKTMN). We approached potential PPI participants via existing groups including the “Sprouting Minds” Young Persons Advisory Group (YPAG) specialising in digital mental health research; the Deep End group, a patient participation group which inputs into study design in order to make research more accessible; and the NIHR Research Design Service (RDS) who offers specialist advice and support on research design and methodology to researchers as well as their own professional contacts from experience working on mental health RCTs.

### Recruitment and data collection

An initial invitation was circulated by email to individuals and groups to share with their members. Those expressing an interest were then sent a copy of the participant information sheet outlining the aim of the study and a consent form. All those who expressed an interest in the study were included.

FGs and interviews were conducted in person, as well as online using Microsoft Teams videoconferencing software. A topic schedule (Additional file 2) was designed to help guide the discussion by eliciting the understanding of the factors that may affect the recruitment approach in clinical trials and how this could impact the inclusivity of the trial participants. We used an exploratory, context-based approach to develop the interview schedule. This included identification of core topics from the literature [21] as a starting point which were then discussed with the project management group who have experience in digital research, design and delivery of online and offline RCTs and equality, diversity and inclusion. In addition, the research team also had input to the interview schedule from two young people PPI with lived experience of mental health issues. The FGs and interviews were conducted between July 2022 and January 2023. The in-person FGs and interviews were audio recorded and the virtual ones were audio and video recorded via Microsoft Office TEAMS, with consent, for later transcription and analysis. Participation was voluntary and participants could withdraw at any time.

### Analysis

An inductive and deductive approach was used to analyse the FG and interview transcripts [35] to develop a coding framework which drew on Braun and Clarke’s thematic analysis [36] in which data are familiarised and coded into themes as part of a review process (see additional file 3). This approach was taken to provide rich data through generation of new codes from the data itself (inductive) as well as identification of codes within the data from known context of the interview schedules [37]. In-person recording was transcribed by KS and Microsoft Office TEAMS produced automated transcripts. Transcript validation was undertaken by two researchers (KS and MI) who read and re-read the transcripts for accuracy and to ensure familiarization with the data. The transcripts were imported into NVivo 12 Pro for coding. The researchers anonymised participants’ information by removing identifying information and allocation of a unique identifier. To ensure validity and reliability of data interpretation, two researchers (KS and MI) independently analysed the transcripts using the topic schedule sections as an initial coding guide and open coding for additional themes. Overall codes were largely consistent between coders, where contradictory coding was apparent, the coders refined this through discussion. Similar codes were clustered together, and a framework of themes identified from the data was developed by KS. The framework was discussed between the research team (KS, MI and CLH) and refined by two researchers (KS and MI) and the final framework was developed when a consensus was reached [36]. The coding process was done in parallel with conducting the FG’s/interviews to inform the interview schedule going forward and to monitor for data saturation. Although we were restricted by available resources to conduct a maximum number of interviews/focus groups, this was not deemed an issue by the interview research team (KS, MI and CLH) and although it can never be certain, it was agreed that no new themes were emerging by the end of the process [38, 39].

## Results

Twenty-three people who volunteered were invited to participate in the study. A total of three FGs and three interviews were conducted with a total of 23 participants (15 PPI partners and eight research staff). Two of the FGs were conducted with 11 PPI partners (three participants in one FG and 8 in the other) and one FG with eight research staff. The three interviews were conducted with four PPI partners. One of these was a joint interview as two participants opted to be interviewed together. Our sample included people who identified as male, female and non-binary as well as representation from white, Asian and Black ethnic groups and were aged between 20 and 70 + years of age.

Four broad themes were identified from the data: [1] recruitment reach; [2] demographic factors that affect selection of recruitment method; [3] safety of technology, and; [4] practical challenges to online recruitment. Supporting quotation represents Staff (Research staff) and PPI (Patients and Public).

### Theme 1 – recruitment reach

Participants commented on three main areas; accessibility and convenience, impact on quality and managing expectations and flexibility.

### Accessibility and convenience

Benefits of using online recruitment methods were their potential for reaching wider communities and greater accessibility to people who may feel stigmatised by their mental health condition or those living with symptoms that may impact their ability to participate.

*“I’d like to say that benefits of online are that you can target quite a wide range of people at once, all at one go” (PPI 11)*.

*“Some groups don’t have good access to like health or social care services so it [online] could help to target different communities.” (PPI 14)*.

*“…it’s [online] more flexible, it allows that better reach as well. We were able to recruit people in [different geographical areas], which otherwise would have been quite a challenge to do, if you had to travel.” (Staff 3)*.

Some PPI and staff participants perceived that online methods could be more convenient for people with mental health conditions and those who care for them.

*“It costs people to travel as well, you know to come to meetings, time and money, or you’ve got kids to pick up, there’s a thousand and one reasons for [online]…” (PPI 3)*.

### Impact on quality and managing expectations

A common view amongst PPI participants and staff was that online methods required closer data monitoring for quality of responders, particularly their eligibility to take part in a trial, where they cited experience of dealing with hoax data or people lying about eligibility to be involved in a study.

*“…we’ve got 1000 hits [social media] and it looks so fantastic and then you whittle it right down and find at the end you’re not that much different to what you got from your clinic. So I would say it works, but it needs a lot of safeguards and in-built procedures, so you can find a way to screen people because people will make up stuff.” (PPI 11)*.

### Flexibility

In order to meet the individual needs of those living with mental health disorders (e.g. condition, age, digital literacy, transport), all participants agreed that offering a range of options was crucial for better recruitment. Participants agreed that researchers should not have a predetermined recruitment method but should explore preferences.

*“…its not one size fits all, so for some individuals they want that distance, they don’t want you there, but for others it works better…others they want that two-way conversation, that contact, that hand on the shoulder, you know showing some empathy, some compassion…” (PPI 2)*.

### Theme 2 – demographic factors that affect selection of recruitment method

Five main factors were identified; age, complexity of mental health, cultural and ethnicity differences, stigma and digital divide.

### Age

PPI participants reported mixed opinions on recruitment preferences for older people. Some stated they would prefer offline methods over online, whereas others noted people are working longer and retiring later and therefore remain proficient in using technology and social media.

*“…you need to build up trust and I think face to face and touch is really important. But that might be because I’m old and I’m not in touch with modern ways…” (PPI 1)*.

*“…people are working longer and longer and they are still using those technologies at work so it will come into their social world.” (PPI 2)*.

PPI participants felt that older people may have limited access to technology and therefore recommended a mixed methods approach to ensure opportunity and inclusivity.

*“Well, I’m an older person, and yes, struggling with technology, but some older people that really don’t access technology at all. So they could be at risk of being missed out. So if you were wanting to target a group of older people, you probably wouldn’t go down the social media route so much, would you? ”(PPI 12)*.

Age was also a factor in PPI and staff views of which media platforms would be most appropriate to use. For instance, Facebook was proposed for those aged 50 + whereas for younger people, TikTok or Instagram were recommended.

*“So if you’re trying to recruit younger people, you probably want to go for Instagram…Facebook, brought in a lot of interest, but I think it does pay to just think about you know lots of different strategies to try and catch a wider variety of population…” (Staff 7)*.

*“You also have to be aware in social media, what the demographics are for social media. So 50 + use Facebook*, Instagram are more younger people, would you use TikTok.*” (PPI 5)*.

### Complexity of mental health and stigma

Both PPI and staff identified the type and stage of mental health condition, as important in the selection of recruitment method. For example, some participants expressed the belief that online methods may reduce the stigma felt by people with mental health disorders through provision of privacy and a virtual space to engage with research from the safety of their own home or safe space.

*“… social anxiety and stuff, they don’t want to talk where somebody can see them, so they might prefer something that was…over the phone, rather than face to face or even on Zoom.” (PPI 4)*.

*“…there were specific groups for posting research and people found that good because some of them had comorbidities that meant they didn’t get out of the house as much to be able to see the research advertisements, and they had social differences that meant that they were less secure coming into a place to do research, so being able to do a lot of it online was a preference.” (Staff 4)*.

However, two key disadvantages to online methods were also acknowledged in relation to the experience of mental health. One, related to vulnerability and online methods, particularly regarding privacy and disclosure of their condition. The second was loss of personal cues, particularly body language was thought to play a significant role in communication in this population.

*“…you’re in a job and somehow it gets out that you have a mental health issue because you’re on a study. Will it get round someway to someone who then shares it with employer or something, and then your vulnerable, aren’t you?” (PPI 15)*.

*“I think that some things can be lost online…we read people’s body language and you know how they are as a person and some of that can be quite difficult to pick up online, you know.” (PPI 11)*.

Participants elaborated on the idea of vulnerability of people living with mental health conditions and a sense of heightened risk of cyber scamming or data fraud as well as potential for online bulling if confidentiality was not maintained appropriately.

*“…there is a stigma against mental health and it’s like, how much do you share that could be accessed by other people, I have been scammed twice. So I realise I am so vulnerable…And if you’re sharing information about someone with a mental health condition, they could be targeted…” (PPI 15)*.

*“So one challenge of recruiting people online…younger people who are living with their families…it might not be safe or appropriate for them to take part online, so it might be better to do that in person.” (PPI 14)*.

### Cultural or ethnic differences

Acceptance and understanding of mental health conditions, as a concept and a clinical disorder, was considered important to encourage engagement in research. For our participants, acceptance was still strongly informed by social and cultural factors which they felt needed to be addressed at a societal level, however they also considered positive generational changes in some cultures were now in evidence.

*“There is that political dimension that almost everyone is aware of, a sense of why is it that a particular profile of say a young Black man with mental health issues and the way he is treated is different to other communities.” (PPI 7)*.

*“I think if you’re talking about mental health there’s still a taboo around mental health and it’s like you tell somebody in an Asian community you’ve got mental health problems, so what’s wrong with your brain what’s wrong with your head…so for that reason people don’t say it.” (PPI 4)*.

Language was still considered as a barrier that could affect recruitment regardless of method used, online or offline.

*“I’m thinking more from the ‘BAME’ community, you know the older generation, they can’t read and write, they can’t go on the internet, if they’re living on their own they wouldn’t know how to get on to Zoom and things like that…” (PPI 4)*.

Trust was raised by several PPI participants as a significant barrier to diversity in research. This was described at a political level and within individual communities that may see themselves as marginalised, e.g. Black African and Black African Caribbean communities. Community gatekeepers were proposed as a way to develop trust within ethnic communities to improve diversity.

*“And we went to the Asian lunch clubs to talk to them…So it is targeting, isn’t it? It’s knowing your community and targeting your delivery really…Rather than throwing something out there and just hoping you’ll get the numbers.” (PPI 15)*.

### Digital divide

There were mixed opinions on access to technology between staff and PPI participants. Whilst staff experience was that the divide was reducing, PPI felt this was still a significant issue for those living in poverty or who were from underprivileged backgrounds including young people. Availability and access to technology was thought to potentially bias recruitment for online methods.

*“We were very worried about the digital devices because you had to have a smartphone to be able to take part. But actually what we’re finding is that…everybody has a smartphone…we’re recruiting parents and even parents from really struggling areas have got a smartphone…” (Staff 6)*.

*“One of the things that concerns me is the assumption that everybody’s got a smart phone or computer. I volunteer…for people with mental health problems and virtually all of them are in a poverty situation and they haven’t got a computer and they find it hard to live day to day and keep a roof over their heads so there’s no way they can afford broad band etc.” (PPI 1)*.

Whether online methods could better reach rural communities than offline methods also produced mixed opinions from participants. For example, lack of infrastructure such as broadband was seen as a disadvantage, however where this was present online methods had the potential to open geographically distanced communities to research opportunities.

The correlation between the pandemic and confidence in using technology such as Zoom was interesting because some participants agreed that post-pandemic, there was a real need and desire for people to reconnect in person. However, others discussed how more people had embraced technology because of the pandemic and now see it as a benefit.

*“…no one would have imagined my elderly parents using YouTube five years ago and now they are quite savvy at using it because they get a lot of benefit out of it [through COVID]. Your mum started using WhatsApp groups.” (PPI 5)*.

*“I think it worked well, particularly because we had to recruit during COVID. And so initially we were going to offer assessments face to face, but we had to take all of that out…most of our consent was online and then all the assessments were online as well…it’s more flexible, it allows that better reach as well. We were able to recruit people in [different geographical areas], which otherwise would have been quite a challenge to do, if you had to travel.” (Staff 3)*.

### Theme 3 – safety of technology

The majority of participants commented on two main areas encompassing confidence and personal interactions.

### Confidence

Although some participants felt people are now more internet aware, issues regarding confidence and security were prominent in the data. All participants agreed that offline methods were considered more trustworthy than online due to existing relationships with health providers and felt it could be hard to ensure the authenticity of online communications. There was contrasting PPI opinions on the safety of data storage, whether people were now more used to providing, holding and sharing data online versus that mistrust and concern over privacy.

*“…you have to think about that ethically, don’t you? It’s OK saying your information is stored and protected, and we look after it but if it’s online, you can’t fully honestly say we have got this secure. Can you? I’m not saying even if it was face to face, you could be secure. But it’s like there’s probably less material that would be shared publicly online.” (PPI 15)*.

*“I don’t think in this day and age, the majority of people…going to be too concerned if they agree to do a study, if they are assured that the personal data is anonymised, that anything that they put in, is logged under a number…” (PPI 9)*.

Participants also felt that older people would be more mistrusting and reluctant to use online methods.

*“…if I look at my daughter and me, I wouldn’t go, for example shopping online…because I don’t know what that site is, whether it’s a trusted site or not, but with the younger generation they don’t really think, they just click…” (PPI 4)*.

Many of the participants felt that, if the trial recruitment wasn’t targeted well or demonstrated trustworthiness and good data protection, it could be viewed as spam or, worse, a phishing email, and therefore just deleted.

*“I don’t open emails because I don’t know who they’re from. There’s a lot of spam, saying click on this and this happened, so unless I know the email, from somebody I know I don’t open them.” (PPI 4)*.

Interestingly, staff participants reported different ways of managing online data safety. Some universities initiated a routine transfer onto the university’s safe haven and clear of website data every day. Other universities used very secure platforms and a strong data management plan, so they don’t have to delete data regularly.

### Personal interaction

Credibility of the recruitment method was an important consideration for PPI participants. The source of the contact had to be recognisable, perceived as trustworthy and safe. Offline methods were deemed safe since they were typically through direct contact with what were perceived as a “trusted” individual such as a health professional, whereas online methods were seen as less so.

*“If you e-mail asking me to join a trial, it’s in spam, I’m far less likely to take any notice which takes us all the way back to in the clinic [in-person], somebody who is appropriately dressed, talking appropriate language…” (PPI 9)*.

*“I think it does help coming from somebody that you know, someone that you trust, because I think you feel more secure in taking part. Whereas online there might be some uncertainty”. (PPI 14)*

In their discussions surrounding trust, both staff and PPI participants stated that the participant/researcher relationship could be built up more easily using offline methods compared to online and if using online then a follow-up contact via telephone, or video conferencing or in-person, was still considered important for retention in RCTs and to build rapport and trust with participants.

*“…creating the rapport with the families is very important and that blended approach…I’m seeing that in the trial we’re doing right now, it’s really, really good and I don’t think it would work as well if you just did everything online.” (Staff 6)*.

*“I think it can be quite impersonal online. So it might help to build a better report if it’s if it’s not online.” (PPI 15)*.

### Theme 4 – practical challenges to online recruitment

Practical challenges emerged mainly in relation to staff knowledge and experience, organisational support and technical considerations.

### Staff knowledge and experience

Experience of the wide range of digital platforms (e.g. Facebook, Twitter, Instagram, TikTok) in a research setting was limited, with staff participants citing lack of training and awareness as a significant issue, particularly around participant safety and data protection. Collection of individual data using digital platforms informing eligibility in the recruitment phase was of specific interest.

*“I have to say, when we started this, none of us had in the trial team had any experience online advertising. So it was quite a steep learning curve…” (Staff 7)*.

### Organisational support and funding

Appropriate resourcing including funding, staff time and organisational support were highlighted as essential. A lack of understanding as well as fear of digital safety at the organisational level to engage with social media platforms were barriers to the use of digital platforms.

*“I do find our clinical trials unit is hyper cautious about everything like that [collecting data], to the point sometimes of being a bit debilitating. It’s almost like you’re not allowed to store any data anywhere.” (Staff 7)*

*“One of the most complicated things we had was how to pay for it. You know, we work in a university. How on earth do you pay Facebook?” (Staff 7)*.

#### Technical considerations

For online communication (via Facebook, Twitter, emails) in terms of information, conciseness, presentation and empathy, are key factors. Participants felt this style of communication, due to its typically limited capacity, should have a clear purpose and be from a respected source to address trust and safety issues. There was also concern from PPI participants that sometimes digital communication could be seen as a bombardment of information which could just get lost.

*“We can’t absorb all this stuff that’s thrown at us and it’s just thrown at you in a way, isn’t it? It [emails] sort of comes at you and, you know, uninvited…” (PPI 15)*.

## Discussion

This study set out with the aim of exploring the perspectives of key partners on the use of online recruitment methods and comparing their pros/cons with offline ones in mental health RCTs.

### Summary of principle findings

Four key themes were identified from the data analysis including recruitment reach, demographic factors that affect selection of recruitment method, safety of technology, and practical challenges to online recruitment. Overall, the study findings indicated the need to offer a flexible and multifaceted approach to participant recruitment to support trial teams to recruit a participant population that is reflective of the people that stand to benefit from the intervention being tested.

### Recommendations for recruitment practices

#### Using a range of recruitment methods could improve inclusivity by expanding opportunities for participation

The study participants overall identified the importance of using both online and offline methods in parallel in recruiting individuals into mental health RCTs. This corroborates the idea of Dawson et al., who recommended the use of an inclusive approach to improve recruitment and retention in RCTs [40]. Whilst integrating online methods is seen as progressive, there are still key challenges researchers should consider, for example online methods are attractive to reach wide geographical populations to improve inclusivity [41], however, it is crucial to consider the risk of losing sight of who is responding [42]. In addition, we found implications for resource allocation to manage digital platforms, the need for well-defined monitoring and screening processes and management of individual expectations in terms of eligibility.

#### Selection of methods should be based on your target participant population

It was agreed that one size does not fit all as an approach to recruitment. People with mental health issues, of all ages, and their carers where present need methods that support their situation both mentally and physically and offer convenience. This finding is consistent with that of a systematic review which found that researchers needed to consider potential participants’ preferences and beliefs that could influence both health provision and willingness to participate in RCTs [43].

Age was a significant factor for consideration of recruitment methods. Although technology was historically seen as a barrier for older people, the problem is likely to reduce with greater generational exposure to technology (smartphones, social media, etc.) [44]. It was clear from the findings that stereotypes of age and technology should be avoided, both for older and younger people and that access to technology remains a significant barrier across all age ranges, specifically affording technology and access to WiFi. Therefore, to be age inclusive a multi-method approach may be preferable and is at present consistent with the literature [19].

Severity of mental health issues and confidentiality has previously been identified as a barrier to participation in mental health research [45–47]. This study found that it is crucial to use an individualised approach where researchers should consider the type of mental health issue, its stage, participants’ feelings and carers’ responsibilities when selecting recruitment methods. For example, using face-to-face methods with people who would benefit from direct contact (low mood) and using online ones for people with conditions where face-to-face interaction might be more challenging (anxiety, obsessive thinking, autism). Using a multi-method approach to recruit people with mental health issues may therefore improve inclusivity and representation. This suggestion is in accordance with other studies indicating that using a balanced recruitment approach is more effective in attracting people to participate in RCTs [40, 48].

There is difficulty in recruiting people from certain ethnic communities who remain underrepresented in mental health research, threatening the generalisability of the trial results and health equality. A strong relationship between cultural/ethnic background and poor participation in mental health research has been reported in the literature [47, 49]. The stigma attached to mental health experienced by ethnic minorities, such as South Asian communities and Black African and Black African Caribbean communities, was perceived to be a strong barrier to participation in mental health research. This stigma has been widely researched and proven to be a factor affecting recruitment, engagement and trust in mental health services and research [47, 50]. Attempts to address stigma and mistrust may increase recruitment. Some participants suggested building relationships with trusted groups and community centres was deemed crucial to facilitate recruitment and achieve ethnic diversity. Community outreach work as well as identifying trusted individuals in these communities have been found to enhance recruitment and engagement efforts [51].

Although some participants mentioned that using the internet has become more ‘normalised’ due to the impact of the COVID pandemic, some people with mental health issues still do not have access to the internet (first-level digital divide) and some do not have the skills to use it (second-level digital divide) [23]. According to recent research at the University of York, people with severe mental health conditions, such as schizophrenia or bipolar disorder, were more likely to lack digital skills and were at greater risk of social isolation due to the digitalisation of health and social care services and research [52].

Digital poverty was raised as a factor affecting recruitment in this study, interestingly opinions were divided between our staff and PPI participants. Staff felt that smartphone access was less of an issue based on their recent experience recruiting successfully to mental health trials. This perspective may also be reflected in the evidence that suggests in 2021 in the UK 88% of all adults had access to a smartphone [53]. When broken down by age, 96% of those aged 16–24 owned a smartphone device compared to 78% aged 55 and above. However, our PPI participants from their own experience working with community and youth groups indicated economic poverty including digital poverty was still an issue. This evidence suggests that to be inclusive, methods of recruitment in RCTs should be varied, accessible and not discriminatory based on access to technology.

#### Online methods should be safe for participants

In recent years, especially post-pandemic, it has been reported that wider society has become more internet aware and trusting of digital platforms through necessity. However, in our study, predominantly our PPI participants felt that there remained an element of fear and scepticism in using online methods compared to offline methods, which they described as more trusted and secure. This is despite evidence suggesting participants being potentially better protected [54]. It is therefore recommended that where online methods are to be used, information on data protection is clear, concise, and readily accessible.

Within our sample we found that vulnerability and discomfort sharing personal information could be a disadvantage of online methods because of concerns regarding confidentiality and anonymity [45, 55]. However, this was offset against the advantages of in-person contact which provided opportunities to read body language and person cues deemed important to support mental health populations during the recruitment process. Verbal and nonverbal communication has a vital role in the process of meaning generation through understanding body posture, tone of voice, and rate of speech. This in turn builds trust and motivation required for participants to understand the aim of the intended trial and improve recruitment [56]. Our staff participants also suggested a range of amelioration strategies that match those observed in other studies recommending the implementation of strict screening procedures and the use of restrictive software features for online methods [57, 58].

Several studies have noted and how mistrust may negatively affect willingness to participate in research [9, 59]. It is interesting to note that in our study there were concerns about solely using online methods, as offline methods were described to give participants that feeling of trust, comfort, engagement and empowerment where people can ask questions. In addition, online methods were considered a limitation for personal interaction thereby potentially reducing understanding and engagement. This is in line with Balfe et al., (2012) who found that use of online recruitment methods could alter the researcher-participant relationship and bring challenges in creating and maintaining engagement and trust between researchers and participants [60].

#### Ensure the recruitment methods selected are appropriately resourced and staff adequately trained

Staff knowledge, organisational support and technical considerations were the most common challenges discussed by the staff participants. Several reports have shown that researchers could inadvertently contribute to underrepresentation through lacking the experience and skills required to recruit and engage a diverse population [61, 62]. This highlights the urgent need for training researchers on various recruitment methods to improve the quality of the included sample, the inclusivity of RCTs and the generalisability of data.

Despite advances in online recruitment methods, technology of pre-screening and initiatives to train researchers, many of these advances will remain underutilised without enough resources. Constrained or low funding remains a barrier to the use of online methods or social media platforms to safely and effectively recruit potential participants [58].

Although the process of targeting potential participants using online methods is considered easy, time-efficient and cost-effective [18], a major challenge still exists of how to differentiate trustworthy and legitimate recruitment invitations from spam and fraudulent misinformation on the internet [63].

### Strengths and limitations

To the best of our knowledge, this is the first study to explore from the perspective of key partners, the advantages and disadvantages of online and offline recruitment methods in RCTs involving mental health populations. This study is grounded in the views and experiences of patients, the public and clinical researchers who work in trial design, conduct and delivery. Although we had input from our experienced multidisciplinary team and two young PPI partners to develop the topic schedule, we acknowledge that having input from a wider group of PPI would have been preferable, but due to funding restraints this was not possible. Another limitation of this study could be the relatively small number of participants, especially in the research staff group, however, the purposive sampling and the diversity in the study sample that included various partners, age groups, ethnicity and genders, could mitigate this risk. We also acknowledge that respondent validation checks were not sought from participants for interview/focus group transcripts due to time and resource constraints although recognised to improve validity in qualitative research. The results of the research have however been shared with those who participated in the interviews and focus groups to which we have encouraged comment to inform future research in this area. Lastly, there may be some drawbacks of having two coders from the same research team. However, interrater reliability varies widely depending on the pair of coders [64] and in this study this may have been mitigated by the different backgrounds of the two coders and there experience in qualitative research and thematic analysis.

## Conclusion

This qualitative sub-study aimed to explore the experiences of key partners on the use of online and offline recruitment of participants into mental health RCTs. Despite perceptions that COVID-19 may have increased the use of digital technology across age and population groups the major finding of this study was the general agreement of using hybrid, balanced and targeted recruitment approaches. As a result, this study highlighted the importance of integrating online with offline methods and considering the preferences of the population under study and their carers, the type of mental health issue and its severity, which would enhance recruitment and inclusivity in mental health RCTs. The findings from this study will be used to develop evidence and considerations for researchers designing RCTs to improve recruitment and engagement in mental health research.

### Electronic supplementary material

Below is the link to the electronic supplementary material.

Supplementary Material 1

Supplementary Material 2

Supplementary Material 3

## Data Availability

Data are available on reasonable request. The unpublished data used and/or analysed during the current study are available from the corresponding author on reasonable request.

## Abbreviations

BAME: Black Asian and minority ethnic
COREC: The consolidated criteria for reporting qualitative research
CRN: Clinical Research Network
EDI: The Equality, Diversity and Inclusion
FG: Focus group
NIHR: The National Institute for Health Research
PPI: Patient and Public Involvement
RCT: Randomised controlled trials
RDS: Research Design Service
RE-MIND: REcruitment in Mental health trials: broadening the ‘net’, opportunities for INclusivity through online methoDs
UKTMN: The UK Trial Managers Network
YPAG: Young Persons Advisory Group
