# Supplementary material for: Widening participation – recruitment methods in mental health randomised controlled trials: a qualitative study
Source: BMC Med Res Methodol. 2023 Sep 21;23:211. doi: 10.1186/s12874-023-02032-1 (PMC10512591; doi:10.1186/s12874-023-02032-1)
Supplement: Supplementary file 2 — Supplementary Material 2 [file 12874_2023_2032_MOESM2_ESM.docx]

**Additional File 2 - RE-MIND Codebook**

(Codebook produced in NVivo 12 Pro)

| **Parent node** | **Child nodes** |
| --- | --- |
| Attitude or stigma |  |
| Benefits of online methods | Better retention or data collection  Inclusive |
| Factors influencing selection of recruitment method | Participant population age  Complexity of needs  Confidence or vulnerability of population  Cultural or ethnic differences  Digital divide/digital poverty  Individual differences/preferences e.g. Physical disabilities or caring responsibilities  Language  Mental health condition  Type of trial  Pandemic  Need for personal interaction/build trust  Staff training  Practicalities or convenience  Rural or geographical communities  Safety of technology or safeguards  Time  Working populations |
| Practical challenges to using online methods | Language translation  Loss of interaction - retention rates  Potential for misunderstanding  Quality of data  Time consuming  Too generic |
| Alternative methods/solutions | Methods should be population/condition appropriate  Be convenient/efficient and supportive – participants and staff  Offer flexibility or choice to reach wider population  Personal follow-up to tech approach  Engage trusted community leads  Interpreters |
| Technical challenges | Accessibility (digital divide/poverty)  Safety and security |
