# Supplementary material for: Widening participation – recruitment methods in mental health randomised controlled trials: a qualitative study
Source: BMC Med Res Methodol. 2023 Sep 21;23:211. doi: 10.1186/s12874-023-02032-1 (PMC10512591; doi:10.1186/s12874-023-02032-1)
Supplement: Supplementary file 3 — Supplementary Material 3 [file 12874_2023_2032_MOESM3_ESM.docx]

**Additional file 1: The consolidated criteria for reporting qualitative research (COREQ)**

| **Domain 1: Research team and reflexivity** |  | | Location in manuscript | |
| --- | --- | --- | --- | --- |
| **Personal Characteristics** | | | | |
| 1. Interviewer/facilitator Which author/s conducted the interview or focus group? | MI, KS and CLH | | Study design, 6. | |
| 2. Credentials  What were the researcher’s credentials? E.g. PhD, MD | KS - MSc  MI-PhD  CLH-PhD | | - | |
| 3. Occupation  What was their occupation at the time of the study? | KS- Assistant Professor CLH- Principal Research Fellow  MI- Research Fellow | | - | |
| 4. Gender Was the researcher male or female? | Three females | | - | |
| 5. Experience and training  What experience or training did the researcher have? | Both MI and KS have previous experience in facilitating and publishing research using focus groups and qualitative methods and CLH and KS have extensive experience in mental health research. | | - | |
| **Relationship with participants** | | | | |
| 6. Relationship established  Was a relationship established prior to study commencement? | Yes | | - | |
| 7. Participant knowledge of the interviewer  What did the participants know about the researcher? e.g. personal goals, reasons for doing the research | Participants were briefed on the purpose and aim of the study which was to explore their perspectives on the use of online and offline methods to recruit trial participants. Ethical approval was obtained for the study and two PPI partners reviewed the participant information sheet and consent form provided their informed consent before starting the focus group or the interview. | | Methods- 6-8. | |
| 8. Interviewer characteristics What characteristics were reported about the interviewer/facilitator? e.g. Bias, assumptions, reasons and interests in the research topic | KS and CLH are experienced in mental health research who might be a potential source of bias. However, both were only facilitating without introducing ideas or opinion that could influence other participants’ perspectives. No other interviewer-related biases identified. | |  | |
| **Domain 2: study design** | | | | |
| **Theoretical framework** | | | | |
| 9. Methodological orientation and Theory  What methodological orientation was stated to underpin the study? e.g. grounded theory, discourse analysis, ethnography, phenomenology, content analysis | Inductive and deductive coding with thematic analysis. | | Methods – 8. | |
| Participant selection | | | | |
| 10. Sampling  How were participants selected? e.g. | Recruited via email. | | Methods – 7. | |
| 11. Method of approach How were participants approached? e.g. face-to-face, telephone, mail, email | Email | | Methods- 7. | |
| 12. Sample size How many participants were in the study? | 23 | | Results – 8. | |
| 13. Non-participation How many people refused to participate or dropped out? Reasons? | The 23 respondents that were invited for focus groups and interviews, all were able to participate. No participant withdrew consent or dropped out. | | - | |
| **Setting** | | | | |
| 14. Setting of data collection  Where was the data collected? e.g. home, clinic, workplace | | Data was collected via the Teams platform and in person. The in-person interview with the Deep End PPI group was conducted at the University of Sheffield International College. The group did not have their own meeting space but used university premises for meetings. | | Methods- 7. |
| 15. Presence of non-participants  Was anyone else present besides the participants and researchers? | | No | | - |
| 16. Description of sample  What are the important characteristics of the sample? e.g. demographic data, date | | Our sample included people who identified as male, female and non-binary as well as representation from white, Asian and Black ethnic groups and were aged between 20 and 70+ years of age. | | Results- 8. |
| **Data collection** | | | | |
| 17. Interview guide Were questions, prompts, guides provided by the authors? Was it pilot tested? | | We used a topic schedule to facilitate focus group and interview discussions. | | Methods – 7. |
| 18. Repeat interviews  Were repeat interviews carried out? If yes, how many? | | No | | - |
| 19. Audio/visual recording  Did the research use audio or visual recording to collect the data? | | The focus groups and interviews were audio (in-person) and/or video (TEAMS) recorded. | | Methods – 7. |
| 20. Field notes  Were field notes made during and/or after the interview or focus group? | | Field notes were also used to record discussions and agreement during FGs and interviews. | | - |
| 21. Duration What was the duration of the interviews or focus group? | | The duration was between 40- 90 minutes. | | - |
| 22. Data saturation  Was data saturation discussed? | | Data saturation was monitored by KS, MI and CLH during the process of data collection. Although we were restricted by available resources to conduct a maximum number of interviews/focus groups, this was not deemed an issue by the research team and although it can never be certain, it was agreed that no new themes were emerging by the end of the process. | |  |
| 23. Transcripts returned Were transcripts returned to participants for comment and/or correction? | | No | | - |
| **Domain 3: analysis and findings** | | | | |
| **Data analysis** | | | | |
| 24. Number of data coders  How many data coders coded the data? | | Two | | Methods- 8. |
| 25. Description of the coding tree  Did authors provide a description of the coding tree? | | Coding tree is provided in Additional file 2. | | - |
| 26. Derivation of themes  Were themes identified in advance or derived from the data? | | Both, the interview schedule provided core topics for discussion to answer the research question. In addition we also identified emerging themes not previously considered in the interview schedule. | | Methods – 8. |
| 27. Software What software, if applicable, was used to manage the data? | | NVivo 12 Pro. | | Analysis – 8. |
| 28. Participant checking  Did participants provide feedback on the findings? | | No | | - |
| **Reporting** | | | | |
| 29. Quotations presented Were participant quotations presented to illustrate the themes / findings? Was each  quotation identified? e.g. participant number | | Yes, specific comments were supported with direct quotes attributed to anonymised participant by role (PPI, YP PPI or Staff) | | Results – 8-22. |
| 30. Data and findings consistent  Was there consistency between the data presented and the findings? | | Yes | | - |
| 31. Clarity of major themes  Were major themes clearly presented in the findings? | | Yes | | - |
| 32. Clarity of minor themes  Is there a description of diverse cases or discussion of minor themes? | | No | | - |
